# Saliva/serum ghrelin, obestatin and homocysteine levels in patients with ischaemic heart disease

**DOI:** 10.5830/CVJA-2016-075

**Published:** 2017

**Authors:** Nermin Kilic, Necati Dagli, Suleyman Aydin, Fazilet Erman, Yuksel Bek, Okhan Akin, SS Kilic, Haci Kemal Erdemli, Hasan Alacam

**Affiliations:** Department of Medical Biochemistry, School of Medicine, Ondokuz Mayis University, Samsun, Turkey; Department of Cardiology, School of Medicine, Firat University, Elazig, Turkey; Department of Medical Biochemistry, School of Medicine, Firat University, Elazig, Turkey; Department of Medical Biochemistry, School of Medicine, Firat University, Elazig, Turkey; Department of Biostatistics, School of Medicine, Ondokuz Mayis University, Samsun, Turkey; Biochemistry Laboratory, Kecioren Education and Research Hospital, Ankara, Turkey; Department of Infectious Diseases and Microbiology, Training and Research Hospital, Samsun, Turkey; Department of Medical Biochemistry, Corum Training and Research Hospital, Corum, Turkey; Department of Medical Biochemistry, School of Medicine, Hacettepe University, Ankara, Turkey

**Keywords:** saliva, homocysteine, ghrelin, obestatin, ischaemia

## Abstract

**Background:**

We aimed to compare ghrelin, obestatin, homocysteine (Hcy), vitamin B_12_ and folate levels in the serum and saliva of ischaemic heart disease patients.

**Methods:**

Serum and saliva were collected from 33 ischaemic heart disease (IHD) patients and 28 age- and body mass index-matched healthy individuals. Levels of acylated and desacylated ghrelin, obestatin and Hcy were determined using the ELISA method.

**Results:**

Acylated ghrelin, desacylated ghrelin and obestatin levels in the saliva were found to be higher than those in the serum of the control group, while acylated and desacylated ghrelin levels in the saliva were significantly lower than those in the serum. Obestatin levels were higher in IHD patients (p = 0.001). Saliva and serum vitamin B12 and folate levels in IHD patients were significantly lower than in the control group (p = 0.001).

**Conclusions:**

It was determined that serum ghrelin levels increased in ischaemic heart disease patients, while serum levels of obestatin decreased.

## Background

Ischaemic heart disease (IHD) is the leading cause of death in developing countries around the world.^1^ It is characterised by atherosclerosis, endothelial dysfunction, lipoprotein oxidation, leukocyte infiltration, the release of various chemotactic and growth factors, and accumulation of cholesterol, lipid and calcium. Fatty streaks cause atherosclerotic plaques, lipid accumulation, and acute and chronic luminal obstruction. The resulting constriction of arteries leads to lack of sufficient blood and oxygen supply to the target organs, which results in ischaemia and necrosis in these organs.^2^,^3^ Hormonal changes are the main alterations that occur in ischaemic heart disease.^4^

Recent studies have reported that ghrelin, the peptide hormone, plays a host of physiological roles in the cardiovascular system. Ghrelin, an endogenous ligand for the growth hormone secretagogue receptor, is synthesised as a pre-prohormone and then proteolytically processed to yield a 28-amino acid peptide.^5^ This peptide was reported to induce growth hormone release; a wealth of evidence, however, has indicated many other physiological activities of ghrelin, including regulation of food intake and energy balance as well as of lipid and glucose metabolism. Ghrelin receptors have been detected in the hypothalamus and the pituitary, but also in the cardiovascular system, where ghrelin exerts beneficial haemodynamic activities.^6^

Ghrelin has been found to exert protective effects on the cardiovascular system.^7^ These include inhibition of vascular endothelial cell apoptosis,^8^ promotion of angiogenesis,^9^ improvement of endothelial dysfunction, enhancement of endothelial nitric oxide synthase (eNOS) expression,^10^ reduction of pro-inflammatory reactions in human endothelial cells, and suppression of vascular inflammation.^11^

The n-octanoylation at serine-3 is critical for ghrelin’s activities. Previous studies have demonstrated that desacylated ghrelin (D-ghrelin), unlike the standard form, does not inhibit tumour necrosis factor-alpha (TNF-α)-induced interleukin-8 (IL-8) release.^11^,^12^

It was reported in another study that obestatin, which is encoded by the same gene as ghrelin, has a regulatory function in the cardiovascular system.^8^ Ghrelin is orexigenic, whereas obestatin is anorexigenic; the former regulates body fluid homeostasis, food intake and energy metabolism, while the latter seems to induce the opposite effects.^13^

The aetiology of endothelial injury relates to many factors, including hyperlipidaemia, hypertension, diabetes mellitus, cigarette smoking and various infectious agents. Elevated homocysteine (Hcy) levels have also proven to be an underdiagnosed cause of endothelial dysfunction. Hcy is a sulphur-containing amino acid involved in two metabolic pathways, catalysed by cystathionine-β- synthase and methionine synthase, depending on vitamin B_6_, B_12_ and folate levels and enzymatic activity of methylenetetrahydrofolate.^14^

Previous studies demonstrated that Hcy decreased endotheliumdependent vasorelaxation and eNOS reactivity, causing endothelial dysfunction in the porcine carotid and coronary arteries.^15^,^16^ Individuals with elevated Hcy levels are therefore at increased risk of atherosclerosis and cardiovascular disease.^17^-^19^

It is not clear whether ghrelin protects vascular tissue from injury that is secondary to risk factors such as high levels of Hcy. Levels of vitamin B^12^ and folic acid, which form part of Hcy metabolism, are in inverse correlation with the total Hcy level. Nutritional deficiency or insufficiency of these vitamins increases the risk of hyperhomocysteinaemia.^20^,^21^

Saliva, which is among the biological fluids used in the diagnosis of diseases, is also used to monitor treatment. Companies that manufacture devices for diagnostic purposes allocate a significant share to research and development efforts geared towards the measurement of multiple parameters in the saliva. This is because saliva is the most important biologicalfluid that could be an alternative to using blood for analysis.^22^

This study aimed to compare ghrelin, obestatin, Hcy, vitamin B_12_ and folate levels in the serum and saliva samples of ischaemic heart disease patients and those of healthy individuals.

## Methods

Patients who had acute coronary syndrome or types 1 and 2 diabetes mellitus, were extremely obese [body mass index (BMI) > 35 kg/m^2^], or had undergone invasive revascularisation in the previous three months were not included in the study. Additionally, individuals were excluded if they were found to have a normal coronary anatomy on angiography but had signs of heart failure and branch blocks on electrocardiography.

The study was carried out on 33 patients (female/male: 48.5/51.5%) with IHD and 28 BMI- and age-matched healthy subjects (female/male: 45.5/54.5%). All subjects, including the controls, underwent coronary angiography. IHD was defined as a ≥ 50% diameter stenosis, as diagnosed by angiography. The control group was defined as those having normal coronary arteries, as diagnosed by angiography. Written consent was obtained before the study, together with the institutional ethics committee’s approval of the study protocol (dated 29 August 2007, issue no. 13).

Hypertension, family history of IHD and smoking were present in 48.5, 36.4 and 33.4% of patients, respectively, in the IHD group. In the control group, 25, 21.4 and 21.4% of patients demonstrated these risk factors. None of the healthy controls had a family history of obesity or a history of abdominal surgery or gastrointestinal disease. They had taken no medication for at least seven days before sample collection.

All subjects were advised not to eat, smoke or drink (except water) during the night before the saliva and blood sample collection. Approximately 2 ml of saliva and 5 ml of blood were taken from the enrolled subjects by the standard procedure described by Hosoda et al.^23^

Total and acylated ghrelin levels in the serum and saliva samples were measured using an enzyme linked immunosorbent assay (ELISA) kit (Linco Research). As desacylated ghrelin level was calculated by subtracting the acylated ghrelin value from the total ghrelin value, care was taken to use the same commercial kits throughout the study.

Serum and saliva obestatin levels were determined using a human obestatin enzyme immunoassay (EIA) kit made by the Bachem brand (Peninsula Laboratories, LLC: a member of the Bachem group, California, USA). Serum and saliva Hcy levels were measured using an axis homocysteine EIA kit. Levels of vitamin B12 and folate were determined by a Roche Elecsys 2010 hormone analyser.

## Statistical analysis

The data were statistically analysed using the SPSS for Windows 15.0 software package. First, continuous variables were checked for normality. Upon finding deviations from a normal distribution, the Mann–Whitney U-test was used to compare the groups. Correlations between blood and saliva values were established by calculating the Spearman correlation coefficient. Mean ± SD and median (min, max) values are presented as descriptive statistics. Frequencies and percentage values are presented for the categorical data. Pearson’s chi-squared continuity correlation analysis was used in the comparison. A p-value < 0.05 was accepted as significant.

## Results

While the age, BMI, diastolic blood pressure, and triglyceride, total cholesterol and low-density lipoprotein (LDL) cholesterol levels were not significantly different between the control and IHD groups (p > 0.05), systolic blood pressure (p = 0.019) and high-density lipoprotein (HDL) cholesterol (p = 0.042) were significantly lower in the IHD group (Table 1).

**Table 1:** Demographic characteristics, biochemical data and serum and saliva levels of biochemical parameters of the controls and patients with ischaemic heart disease

**	*Control*		*Control IHD*		**
**	*Mean ± SD*	*Med (min; max)*	*Mean ± SD*	*Med (min; max)*	*p-value*
Age 49.5 ± 12.2 48.0 (25; 77) 50.5 ± 11.0 49.1 (24; 75) 0.126	49.5 ± 12.2	48.0 (25; 77)	50.5 ± 11.0	49.1 (24; 75)	0.126
BMI (kg/m^2^)	25.1 ± 3.1	25.6 (20.3; 32.8)	25.5 ± 3.4	26 (21.4; 33.8)	0.860
Systolic blood pressure (mm/Hg)	116.8 ±10.1	120 (90; 140)	126 ± 14.4	120 (100; 160)	0.019
Diastolic blood pressure (mm/Hg)	74.0 ± 7.6	70 (60; 90)	75.8 ± 70.1	80 (60; 90)	0.488
Triglycerides (mg/dl)	179.3 ± 77.4	153 (83; 340)	169 ± 86.5	157 (73; 491)	0.633
[mmol/l]	[2.03 ± 0.87]	[1.73 (0.94; 3.84)]	[1.91 ± 0.98]	[1.77 (0.82; 5.55)]	
Total cholesterol (mg/dl)	177.1 ± 24.7	172 (140; 210)	194 ± 57.4	191 (120; 369)	0.128
[mmol/l]	[4.59 ± 0.64]	[4.45 (3.63; 5.44)]	[5.02 ± 1.49]	[4.95 (3.11; 9.56)]	
LDL cholesterol (mg/dl)	121.2 ± 21.7	126 (81; 152)	130 ± 50.0	126 (73; 270)	0.761
[mmol/l]	[3.14 ± 0.56]	[3.26 (2.10; 3.94)]	[3.37 ± 1.30]	[3.26 (1.89; 6.99)]	HDL cholesterol (mg/dl)
HDL cholesterol (mg/dl)	39.1 ± 6.3	38.5 (3; 54)	35.9 ± 8.7	34 (22; 57)	0.042
[mmol/l]	[1.01 ± 0.16]	[1.00 (0.08; 1.40)]	[0.93 ± 0.23]	[0.88 (0.57; 1.48)]	
Total ghrelin (pg/ml)					
Serum	111.4 ± 34.5	105.5 (49; 169)	316 ± 82.4	311 (229; 627)	0.001
Saliva	205.8 ± 30.8	218 (150; 242)	260 ± 68.1	250 (98; 510)	0.001
Acylated ghrelin (pg/ml)					
Serum	16.9 ± 4.8	16 (10; 29)	53.8 ± 7.9	56 (37; 68)	0.001
Saliva	29.6 ± 5.5	31 (19; 39)	32.7 ± 8.0	320 (18; 45)	0.101
Desacylated ghrelin (pg/ml)					
Serum	93.8 ± 31.4	89.5 (38; 145)	255 ± 67.5	254 (171; 435)	0.001
Saliva	176.5 ± 30.2	189.5 (119; 212)	220.2 ± 43.1	211 (125; 314)	0.001
Obestatin (pg/ml)					
Serum	399.5 ± 83.7	406.5 (100; 503)	371 ± 62.1	347 (303; 499)	0.020
Saliva	541.2 ± 57.3	529 (455; 688)	588 ± 63	597 (482; 692)	0.005
Homocystein (μmol/l)					
Serum	9.4 ± 1.4	9.2 (7.2; 12.6)	14.3 ± 3.7	13.6 (8.4; 26)	0.001
Saliva	1.2 ± 0.2	1.1 (1; 1.5)	1.2 ± 0.1	1.2 (1; 1.5)	0.900
Vitamin B_12_ (pg/ml)					
Serum	255.1 ± 54.4	250.6 (57.3; 330)	175 ± 34.9	162.6 (133; 260)	0.001
Saliva	78.8 ± 10.4	78.5 (63; 98.8)	58.6 ± 7.4	59 (43.8; 80.2)	0.001
Folate (ng/ml)					
Serum	7.3 ±1.3	6.8 (5; 9)	5.1 ± 0.9	4.8 (4.0; 7.1)	0.001
Saliva	4.0 ± 0.5	4.2 (3; 4.8)	2.9 ± 0.8	2.7 (2.1; 5.1)	0.001

p-values obtained by comparison of control and IHD groups.

An intra-group comparison of serum and saliva levels of total acylated and desacylated ghrelin, obestatin, vitamin B_12_ and folate was conducted. These parameters were also compared between the groups. Some differences were seen between serum and saliva levels of the biochemical parameters when we carried out intra- and inter-group comparisons (Table 1).

**Fig. 1.**
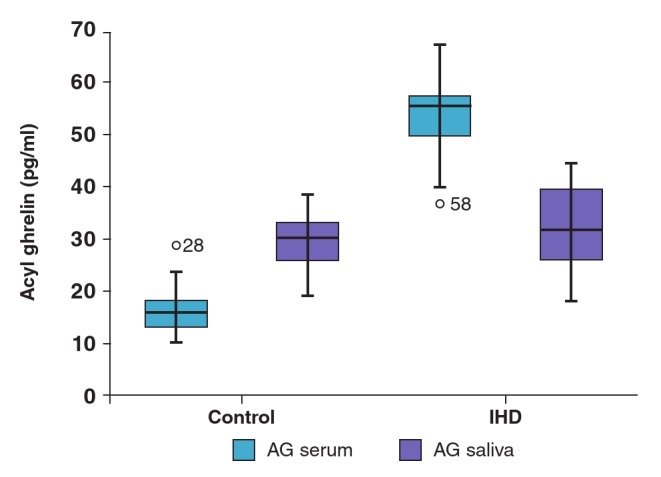
Serum and saliva acylated ghrelin levels of controls and patients with ischaemic heart disease.

In the control group, saliva levels of total acylated and desacylated ghrelin were higher than in the serum (p = 0.001). Conversely, in the IHD group, serum levels were found to be higher than in the saliva (p = 0.001) (Table 1, Fig. 1).

**Fig. 2.**
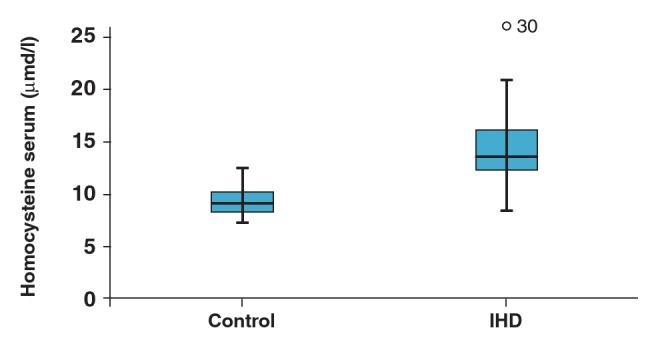
Serum Hcy levels of controls and patients with ischaemic heart disease.

**Fig. 3.**
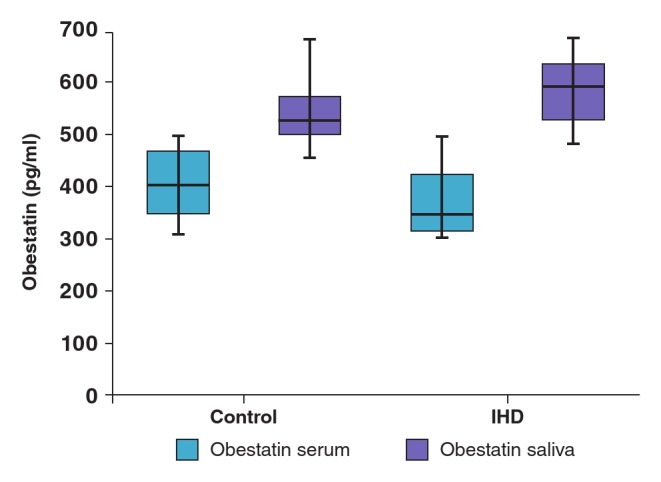
Serum and saliva obestatin levels of controls and patients with ischaemic heart disease.

When serum and saliva levels of these parameters were compared between the two groups, total ghrelin, desacylated ghrelin and serum levels of Hcy and acylated ghrelin were found to be higher in the IHD group (p = 0.001) (Table 1, Fig. 1, 2). While serum levels of obestatin were lower in the IHD group, saliva levels were higher (p = 0.001) (Table 1, Fig. 3). Saliva and serum levels of vitamin B_12_ and folate were significantly lower in the IHD patients in comparison with the control group (p = 0.001) (Table 1).

## Discussion

Ghrelin is a 28-amino acid peptide that was initially identified in rat stomachs.^12^ In humans, it is mainly secreted from the stomach and acts as the endogenous ligand for the growth hormone secretagogue receptor (GHS-R).^24^,^25^ There are two subtypes of GHS-R: GHS-R subtype 1a (GHS-R1a) and subtype 1b (GHS-R1b). GHS-R1a is the functionally active and signal-transducing form.^26^,^27^> It is expressed in many tissues, such as pituitary and thyroid glands, pancreas and cardiovascular tissues, including myocardial and endothelial cells.^9^,^10^,^25^

Ghrelin has two major endogenous forms, acylated ghrelin (A-Ghr) and unacylated ghrelin (DA-Ghr). A-Ghr can bind GHS-R1a and exert biological functions, unlike the unacylated form.^28^

Obestatin is a 23-amino acid peptide that is co-secreted with ghrelin from the stomach.^29^ Although various groups have reported that obestatin is able to reduce appetite, gastric emptying and jejunal motility, and exert proliferative, survival and anti-apoptotic effects in B-cells, its biological effects remain highly controversial and need to be thoroughly investigated.^30^,^31^

In this study, saliva levels of both acylated ghrelin and obestatin were found to be higher than the serum levels in the control group. In the IHD group, however, serum levels of acylated ghrelin and saliva levels of obestatin were higher. In our previous study, we found that saliva levels of both parameters were higher than the serum levels in both groups.^22^

The high ghrelin and obestatin levels found in saliva are very likely a consequence of the function of the salivary gland, since obestatin is produced by this gland. Both our own research team^22^ and Gröschl et al.^32^ have previously independently reported that the salivary gland produces its own ghrelin.

In our previous study,^4^ we attributed the significantly higher saliva levels of ghrelin and obestatin, in comparison to serum levels, to the fact that they had undergone greater degradation in the blood due to pre-analysis errors, such as the temperature of collection and centrifugation. In the present study, when the serum and saliva levels of these peptides were compared between groups, it was found that serum ghrelin levels were elevated while serum obestatin levels were reduced in the patients with IHD, compared to the control group. We believe that these elevated ghrelin levels occur in order to curtail ischaemic heart damage.

In a study conducted by Laurila et al.,^33^ high plasma ghrelin concentration was correlated with protection from coronary heart disease. Sax et al. found that pericardial active ghrelin concentration and the pericardial-to-plasma ghrelin ratio were elevated in IHD patients, compared to non-ischaemic subjects, and suggested an increased ghrelin production by the chronically ischaemic myocardium.^34^ Gnanapavan et al. demonstrated mRNA expression of ghrelin in the myocardium and veins. They determined that GHS-R1a was expressed in the myocardium and not the veins, while GHS-R1b was expressed in both.^25^ On the basis of these studies, it can be asserted that ghrelin is a critical peptide for the cardiovascular system.

Li et al. examined the role of pro-inflammatory cytokines, reporting that ghrelin may inhibit the TNF-α-induced IL-8 release in a concentration-dependent manner.^11^ Mononuclear cell adhesion molecules are an integral part of vascular inflammation and atherosclerosis, as induced by chemotactic cytokines. Ghrelin inhibits the activity of nuclear factor kappa B (NF-κB), which is crucial in the production of chemotactic cytokines, and adhesion molecule expression, which adversely affects endothelial cell response.^11^ Ghrelin has also been shown to improve left ventricular function in heart failure.^35^,^36^

In addition, ghrelin’s mechanism of action on endothelial cells may be linked to GHS-R, a seven-transmembrane G protein-coupled ghrelin receptor. Stimulation of GHS-R with ghrelin leads to activation of G protein, calcium mobilisation and multiple downstream signalling.^37^ Ghrelin receptors have been isolated in various tissues, such as the endocrine glands and cardiovascular tissue. In addition, receptor density changes have been demonstrated to be an important part of the cardiovascular effects of ghrelin.^38^ Ghrelin has also been reported to prevent apoptosis in cardiac cells.^8^

An inverse correlation was found between serum total ghrelin levels and systolic blood pressure in our study. Studies reporting that ghrelin significantly reduced mean arterial blood pressure confirm our results.^35^,^39^,^40^

In this study, both patients and controls had a mean BMI higher than that considered normal worldwide, although they are not regarded as obese. The mean BMI for the patients in the study was 25.5 ± 3.4 kg/m^2^, which is classified as overweight. Therefore, the BMIs of the controls (25.1 ± 3.1 kg/m^2^) were matched to those of the patients. If the BMIs of the controls and patients had not been matched, it may have influenced obestatin and ghrelin secretion independent of the heart disease, as an increase in ghrelin level has been correlated with a decrease in body weight.^41^ A weakly negative correlation was also found between the serum acylated ghrelin and BMI in the IHD subjects, but there was no correlation in the controls. Several previous studies support our findings.^22^,^32^,^41^,^42^

Aydin assumed that low serum levels of obestatin also served to reduce ischaemic damage, as obestatin and ghrelin counteract one another.^13^ However, given that an elevated saliva obestatin level is only related to the circulation via transport from blood to saliva, and that the salivary gland also secretes obestatin, it is presumed that the elevated saliva level may have resulted from the contribution of this gland.^2^

In addition, Iglesias and colleagues recently found that obestatin had no effect on cardiomyocyte viability and metabolism.^43^ The pathophysiological role of obestatin in ischaemic heart disease therefore remains an important research topic.

Salivary Hcy levels in both groups were found to be significantly lower than serum levels in our study. A possible explanation for this is that Hcy, which is a very weak lipophilic molecule, is bound to plasma proteins in large amounts, with the result that its diffusion to saliva is reduced. In routine clinical chemistry, the measured salivary components diffuse into the saliva from the blood. It has been reported that several factors play a part in the diffusion of serum components, such as Hcy, into saliva, namely that lipophilic components diffuse more easily than lipophobic components, the rate of ionised components in the saliva to those in the plasma changes with salivary pH, and the binding of components to proteins is a crucial factor in this rate.^44^

While there was no significant difference between the salivary Hcy levels of the control and IHD groups, serum Hcy levels were found to be significantly higher in IHD patients. One reason why serum Hcy levels increase in IHD is believed to be a deficiency of vitamin B_12_ and folic acid, which function as co-factors and co-substrates in Hcy metabolism.^45^,^46^ Serum levels of B_12_ and folic acid, which act in the pathway of the Hcy metabolism, were also found to be significantly lower in the patient group in our study.

An elevated Hcy level is considered to be a risk factor for the development of atherosclerosis. It has been suggested that Hcy influences endothelial function, leading to a prothrombotic environment, platelet activation, and endothelial leukocyte interactions.^47^ In addition, Hcy enhances inflammatory responses, which are recognised for their role in atherosclerotic disease.^48^,^49^ Recent studies^50^,^51^ suggest that markers of inflammation may reflect different aspects of the atherothrombotic process and have a potential role in the prediction of risk for developing coronary artery disease. Besides the detrimental effects of Hcy on the cardiovascular system,^15^,^52^ elevated Hcy levels are also associated with peripheral arterial disease as well as venous diseases such as deep-vein thrombosis.^53^

Although there is strong evidence to suggest that increased ghrelin levels lead to increased food intake and lipid deposition, its cardiovascular benefits, such as the inhibition of cytokine production and improved left ventricular function, have also been well documented. Ghrelin receptors have been isolated in various tissues, such as the endocrine glands and cardiovascular tissue. In addition, receptor density changes have been demonstrated to be an important part of the cardiovascular effects of ghrelin.^38^ Targeting specific tissue receptors by modification of the ghrelin molecule may achieve the desired cardiovascular effects without activating the unwanted effects of ghrelin.

The mechanism of improvement in endothelial function relates to improved eNOS expression and a reduction in oxidative stress. Ghrelin also has a potent effect on blocking Hcy-induced reduction in eNOS protein levels.^54^ Disproportion in the quantity of reactive oxygen species (ROS) generated during aerobic metabolism is known to lead to oxidative stress and contribute to vascular disease. This process is carried out through a variety of mechanisms, including nitric oxide (NO) consumption and depletion,^55^,^56^ regulation of gene transcription,56 and intracellular alkalinisation.^57^ Ghrelin reduced the production of superoxide anion, a major type of ROS, in Hcy-treated porcine coronary artery and human endothelial cell rings.^54^

## Conclusion

Serum Hcy, serum acylated ghrelin and saliva obestatin levels were significantly elevated, while serum obestatin level decreased in the IHD group. We believe that ghrelin is a potent and effective protein that inhibits Hcy production and other potentially damaging mechanisms and may underlie the decrease in obestatin level, which counteracts ghrelin. It can also be concluded from this study that saliva could be an alternative to serum in the diagnosis and follow up of disease, but these results should be confirmed with larger groups of subjects.
